# Targeted Delivery of Chimeric Antigen Receptor into T Cells via CRISPR-Mediated Homology-Directed Repair with a Dual-AAV6 Transduction System

**DOI:** 10.3390/cimb45100486

**Published:** 2023-09-22

**Authors:** Pablo D. Moço, Omar Farnós, David Sharon, Amine A. Kamen

**Affiliations:** Department of Bioengineering, McGill University, Montreal, QC H3A 0E9, Canada; pablo.moco@mail.mcgill.ca (P.D.M.);

**Keywords:** adeno-associated viral vectors, T cells, genetic engineering, CRISPR/Cas9, homology-directed repair, chimeric antigen receptor

## Abstract

CAR-T cell therapy involves genetically engineering T cells to recognize and attack tumour cells by adding a chimeric antigen receptor (CAR) to their surface. In this study, we have used dual transduction with AAV serotype 6 (AAV6) to integrate an anti-CD19 CAR into human T cells at a known genomic location. The first viral vector expresses the Cas9 endonuclease and a guide RNA (gRNA) targeting the T cell receptor alpha constant locus, while the second vector carries the DNA template for homology-mediated CAR insertion. We evaluated three gRNA candidates and determined their efficiency in generating indels. The AAV6 successfully delivered the CRISPR/Cas9 machinery in vitro, and molecular analysis of the dual transduction showed the integration of the CAR transgene into the desired location. In contrast to the random integration methods typically used to generate CAR-T cells, targeted integration into a known genomic locus can potentially lower the risk of insertional mutagenesis and provide more stable levels of CAR expression. Critically, this method also results in the knockout of the endogenous T cell receptor, allowing target cells to be derived from allogeneic donors. This raises the exciting possibility of “off-the-shelf” universal immunotherapies that would greatly simplify the production and administration of CAR-T cells.

## 1. Introduction

CAR-T cell therapy is a type of immunotherapy that involves the genetic engineering of a patient’s T cells to express a chimeric antigen receptor (CAR) on their surface. This synthetic receptor artificially redirects the specificity of the T cells to tumour cells [1]. Such receptors have an extracellular recognition domain derived from an antibody and can recognize any antigen to which it has an affinity. Their intracellular domain can combine signals from the receptor complex, such as CD3ζ and T cell co-stimulatory molecules [2]. Once the target antigen is recognized, the CAR-T cell is activated, releasing cytokines and enzymes that kill the target tumour cell. CAR-T cells recognize molecules on the surfaces of tumour cells independently of the major histocompatibility complex (MHC), making the antitumour response more effective [3]. This therapy has shown great promise in early clinical trials, leading to its approval by the US Food and Drug Administration for treating certain types of leukemia and lymphoma [1]. The production process, however, can take several weeks and requires specialized equipment and trained personnel [4], leading to expensive therapy, with overall treatment costs surpassing 1 million US dollars [5].

Recombinant adeno-associated viruses (AAVs) are small, non-pathogenic viruses packaging a 4.7-kb-long ssDNA genome that have been extensively studied for their potential use in gene therapy [6]. For the generation of recombinant AAVs, the *Rep* and *Cap* genes, which encode for proteins related to viral genome replication and capsid formation and the assembly of the wildtype AAV, are replaced with the transgene of interest flanked by inverted terminal repeats (ITRs). One of the characteristics of recombinant AAV vectors is their ability to deliver and express transgenes stably without integration into the host genome because these vectors do not retain the *Rep* gene [7]. In most cases, the AAV genome remains in an episomal state, which confers low toxicity and allows for the expression of transgenes for long periods of time in non-dividing cells [8]. However, the lack of integration can also limit the durability and effectiveness of AAV gene therapies. On the other hand, the AAV-delivered ssDNA genome can serve as a template for gene targeting via homologous recombination [9,10,11]. While still not completely understood, this method is considered more efficient than other conventional approaches, such as plasmid transfection. Possible explanations include more efficient nuclear delivery of the transgene, the fact that the AAV genome is single-stranded, and the effect of the ITRs on recruiting the cellular repair machinery [9,12,13,14]. Additionally, the introduction of a double-strand break (DSB) into the host genome by endonucleases improves the frequency of this AAV-mediated recombination [14,15,16,17].

Five types of nuclease proteins can be used to make specific changes to the genome by creating a DSB in the DNA: Zinc-finger nucleases (ZFNs), transcription activator-like effector nucleases (TALENs), homing endonucleases, meganucleases, and CRISPR RNA-guided nucleases such as Cas9. These proteins have been employed to integrate the CAR transgene into T cells at specific sites, as reviewed by Dabiri et al. [18]. The CRISPR/Cas9 system has made it possible to introduce DSBs at specific genomic loci with high precision. This allows for targeting the AAV genome to a specific genomic locus, where it can integrate into the host genome via homology-directed repair. This approach can increase the stability and expression of transgenes delivered by AAVs, such as the chimeric antigen receptor [19]. In the case of CAR-T cell therapies, the use of AAVs and CRISPR/Cas9 can enable the site-specific integration of CAR transgenes into the host genome, leading to more durable and effective CAR-T cells [20]. Moreover, the CRISPR/Cas9 system can knock out endogenous genes in T cells, such as the T cell receptor, to generate “universal” CAR-T cells that are potentially more affordable and accessible [4,21].

Current CAR-T cell products are generated via the transduction of lentivirus (Kymriah, Breyanzi, Abecma, and Carvykti) or γ-retrovirus (Yescarta and Tecartus) due to the achievement of high rates of transduction and long-term expression of the CAR transgene. The use of these vectors is deemed safe [22], but their variable transgene integration [23,24] poses the risk of insertional oncogenesis [25,26,27,28] and of clonal expansion [29]. Additionally, the transgene integration can occur in sites with distinct transcriptional activity, resulting in variable CAR expression and inadequate therapeutic outcomes [30]. Targeting the CAR transgene to the T-cell receptor alpha constant (*TRAC*) locus alleviates these risks and leads to CAR-T cells with increased antitumour efficacy while exhibiting a reduced exhaustion profile, as previously reported [20]. Generating a potential universal CAR-T cell product is another benefit of targeting the *TRAC* [31]. By knocking out the endogenous TCR, the risk of graft versus host disease (GvHD) is eliminated as the recipient’s immune system does not recognize the alloantigen [32,33]. The endogenous MHC can also be knocked out to generate “off-the-shelf” allogeneic CAR-T cells by targeting the beta-2 microglobulin (*B2M*) gene, reducing the risk of graft rejection [32]. In contrast to the current labour-intensive and time-consuming manufacturing process of CAR-T cell products [4], which increases its price and limits its application and availability to patients [5], the manufacturing of universal CAR-T cells has the potential to lower the costs and increase the accessibility of the product to patients whose T cells have been depleted due to cancer or previous treatments [4,34]. Additional targets for knockout can include *PD1*, for the generation of exhaustion-resistant CAR-T cells [35,36], and some target antigens that are also expressed on the surfaces of T cells, such as CD7 and CD33, for the reduction of on-target/off-tumour toxicity [37,38]. Here, we describe an alternative method for the site-specific integration of CAR into T cells via dual transduction with AAV6, a serotype extensively used for homology-mediated transgene integration in different human cells, including induced pluripotent stem cells [39,40], hematopoietic stem cells [17,41,42,43], and, most importantly, the immune cells NK and T cells [20,36,44,45,46,47,48]. The first viral vector encodes for the CRISPR/Cas9 machinery, while the second vector carries the DNA template for transgene integration via homology-directed repair. Simultaneous endogenous TCR and CAR integration disruption could be produced from allogeneic donors as an “off-the-shelf” therapy. This study shows a proof of principle for dual-AAV6-mediated site-specific CAR-T cell generation.

## 2. Materials and Methods

### 2.1. Cell Culture

HEK293SF-3F6 (HEK293SF) cells were kindly provided by the National Research Council Canada. Jurkat cells were kindly provided by Prof. Mohammad-Ali Jenabian (Immuno-Virology Lab, Department of Biological Sciences and CERMO-FC Research Centre, Université du Québec à Montréal (UQAM), Montréal, Québec, Canada). HEK293SF cells were maintained in serum-free suspension cultures at 37 °C, 5% CO_2_, and 75% relative humidity in a shaker incubator (Infors HT, Bottmingen-Basel, Switzerland) at 135 rpm speed of agitation. The medium for both cell maintenance and viral vector production was HyCell TransFx-H (Cytiva Life Sciences, Marlborough, MA, USA) supplemented with 0.1% *w*/*v* of Kolliphor P188 (Sigma-Aldrich, Burlington, MA, USA) and 4 mM GlutaMAX (Gibco, Billings, MT, USA). Jurkat cells were maintained in RPMI-1640 (Cytiva Life Sciences, Marlborough, MA, USA) supplemented with 10% *v*/*v* fetal bovine serum (FBS, Cytiva Life Sciences, Marlborough, MA, USA) and 1% *v*/*v* penicillin–streptomycin (pen-strep, Gibco, Billings, MT, USA) at 37 °C, 5% CO_2_, and 75% relative humidity in a static incubator (Panasonic, Tokyo, Japan).

### 2.2. Guide RNAs and Plasmids

The following guide RNAs targeting the *TRAC* locus were evaluated: (1) ataggcagacagacttgtca; (2) gtctctcagctggtacacgg; and (3) tacacggcagggtcagggtt. Each guide RNA (gRNA) was synthesized as 2 complementary DNA strands with BsaI overhangs at the 5′ end. The oligo DNAs were annealed and inserted into the plasmid pX601-AAV-CMV::NLS-SaCas9-NLS-3xHA-bGHpA;U6::BsaI-sgRNA (a gift from Feng Zhang, Addgene plasmid #61591; http://n2t.net/addgene:61591, accessed on 7 April 2021; RRID:Addgene_61591), which encodes for the SaCas9, via restriction cloning at the BsaI site [49]. The three plasmids containing each of the three gRNAs were named pX601-AAV-SaCas9-TRAC#1, pX601-AAV- SaCas9-TRAC#2, and pX601-AAV-SaCas9-TRAC#3, respectively. The plasmid containing the DNA template for homology-directed repair was designed to encode for the gene of the anti-CD19 chimeric antigen receptor (FMC63-28Z receptor protein) and an EGFP marker based on the plasmid pSLCAR-CD19-BBz [50], flanked by a 150-bp homology arm upstream of the template (left homology arm, LHA) and a 500-bp homology arm downstream of the template (right homology arm, LHA). The ITRs flank the entire construction for packaging into an AAV vector. The construct was synthesized by BioBasic (Markham, ON, Canada) and cloned into a pUC57 vector. The final plasmid was named pUC57_AAV_anti-CD19_CAR_HDR.

### 2.3. Analysis of Double-Strand Break Efficiency

HEK293SF cells were transfected with each of the three plasmids (1 µg/mL) to evaluate double-strand break (DSB) efficiency. Non-transfected cells were used as the negative control. Ninety-six hours post-transfection, the genomic DNA of the cells was extracted using a PureLink™ Genomic DNA Mini Kit (Invitrogen, Waltham, MA, USA), as per the manufacturer’s instruction. The CRISPR/Cas9 target site was amplified via PCR using primers targeting the *TRAC* locus (forward: 5′-GCCAACATACCATAAACCTCCC-3′; reverse: 5′-GGACTGCCAGAACAAGGCTC-3′). The amplicons were sequenced by Sanger sequencing at Genome Québec (Montréal, QC, Canada), and the DSB efficiency was evaluated by Tracking of Indels by Decomposition (TIDE) [51]. The TIDE software (http://shinyapps.datacurators.nl/tide/, accessed on 15 September 2022) utilizes quantitative sequence trace data from two standard capillary sequencing reactions. These sequence traces are subjected to analysis through a custom decomposition algorithm, identifying the major induced mutations (insertions and deletions (indels)) at the intended editing site and quantifying their occurrence within the cell population to determine the overall efficiency.

### 2.4. Viral Vector Production

Adeno-associated viral vectors serotype 6 packaging the SaCas9 and gRNA #1 (AAV6-SaCas9) or the CAR (AAV6-CAR) genomes were produced by triple transient transfection as previously described [52], where the transgene plasmids used were pX601-AAV-SaCas9-TRAC#1 and pUC57_AAV_anti-CD19_CAR_HDR, respectively. After viral harvest, the cell lysate was clarified using a 1.2/0.5-µm Optiscale capsule (Millipore Sigma, Burlington, MA, USA). The clarified lysate was then subjected to single-step affinity capture chromatography using a commercially available 5-mL prepacked immunoaffinity resin column, POROS™ CaptureSelect™ AAVX (ThermoFisher Scientific, Waltham, MA, USA). The affinity purification process was conducted on the ÄKTA Avant25 FPLC system (Cytiva Life Sciences, Marlborough, MA, USA). The affinity-resin-bound AAVs were eluted in 0.1 M glycine (pH 2.5) and immediately neutralized by adding 10% *v*/*v* of neutralization buffer containing 1 M Tris, 20 mM MgCl_2_, and 20% sucrose (pH 8.8). The purified AAV6 vectors were further concentrated using 100-KDa Amicon^®^ Ultra-5 centrifugal filter units (Millipore Sigma, Burlington, MA, USA) and stored at −80 °C. The AAV6 vector titers were determined by ddPCR analysis as previously described [52], using the following pair of primers: 5′-GGCCAGATTCAGGATGTGCT-3′ (forward) and 5′-CATCATCCCCAGAAGCGTGT-3′ (reverse), for AAV6-SaCas9; 5′-GAGGAAACGGGGCAGAAAGA-3′ (forward) and 5′-GGCCTTCCTGAGGGTTCTTC-3′ (reverse), for AAV6-CAR.

### 2.5. Analysis of AAV6-Delivered CRISPR/Cas9

The efficiency of the AAV6-delivered SaCas9 and gRNA #1 to be expressed and cleave the target DNA was evaluated by transducing HEK293SF cells at a multiplicity of infection (MOI) of 10^3^ and co-infection with E1/E3-deleted adenovirus type 5. RNA was extracted from the cells 24 and 48 h post-transduction. The extraction was done using the Aurum™ Total RNA Mini Kit (Bio-Rad, Hercules, CA, USA). cDNA was synthesized using the iScript™ cDNA Synthesis Kit (Bio-Rad, Hercules, CA, USA), per protocol. Oligo(dT) primers were used to prepare the sample for the detection of SaCas9 expression, and gene-specific primers (5′-CGCCAACAAGTTGACGAGAT-3′ and 5′-GGCAGACAGACTTGTCAGTTTTA-3′) were used to prepare the sample for the detection of gRNA expression. The cDNAs were amplified via PCR using primers for SaCas9 (5′-GGCCAGATTCAGGATGTGCT-3′ and 5′-CATCATCCCCAGAAGCGTGT-3′) and gRNA #1 (same pair of primers used for cDNA synthesis). The size of the amplicons was analyzed via agarose gel electrophoresis. At 24 and 48 h post-transduction, samples were collected to detect the expressed SaCas9 protein. Following cell lysis, the samples were run on polyacrylamide gel electrophoresis. After blotting the samples to a nitrocellulose membrane, the membrane was blocked and incubated with 0.5 µg/mL of anti-SaCas9 monoclonal antibody (11C12, GenScript, Piscataway, NJ, USA) overnight. Peroxidase goat anti-mouse antibody (IgG (H + L) Jackson ImmunoResearch, West Grove, PA, USA, RRID:AB_2307346) was used as the secondary antibody. Genomic DNA was also extracted from the cells 48 h post-transfection. As previously described, the *TRAC* locus was amplified by PCR, and the material was sequenced and analyzed via agarose gel electrophoresis and TIDE.

### 2.6. Dual-AAV Transduction of Jurkat Cells

Jurkat cells were resuspended at a density of 0.5 × 10^6^ cells/mL in RPMI supplemented with 10% FBS and 1% penicillin–streptomycin and seeded into wells of a 48-well plate. The cells were transduced with AAV6-SaCas9 and AAV6-CAR at medium and high MOI. For medium MOI, 1 × 10^5^ genome-containing particles (viral genomes, VG) of each vector were added per cell. For high MOI, 2 × 10^6^ VG of AAV6-SaCas9 and 7 × 10^6^ VG of AAV6-CAR were added per cell. The transduced cells were incubated at 37 °C, 5% CO_2_, and 75% relative humidity in a static incubator, as previously described. At 48 h post-transduction, the cells for the high MOI group were resuspended in a fresh medium.

### 2.7. Genomic Analysis of Modified Jurkat Cells

Extraction of genomic DNA from up to 5 × 10^5^ Jurkat cells was done at 48 and 96 h post-transduction for the medium and high MOI groups, respectively. Non-transduced cells were used as controls. Genomic DNA was extracted as described above and used as a template for PCR using primers for the *TRAC* locus. The size of the PCR products was analyzed by agarose gel electrophoresis, and the amplicons were sequenced for TIDE analysis. Quantitation of gel bands was done using Image Lab 6.1 (Bio-Rad, Hercules, CA, USA) using a 1-kb Plus DNA Ladder (New England BioLabs, Ipswich, MA, USA) as a standard curve. Purified PCR products were used for the evaluation of the site-specific insertion of the transgene. The PCR reaction contained one primer positioned upstream of the 3′ junction (TRAC-Forward: 5′-GCCAACATACCATAAACCTCCC-3′) and one downstream, inside the transgene (CAR-Reverse: 5′-GGCCTTCCTGAGGGTTCTTC-3′). The PCR product was run on an agarose gel, and the 2.2-kb band was extracted using the QIAquick Gel Extraction Kit (QIAGEN, Germantown, MD, USA). The extracted DNA was analyzed by Sanger sequencing and enzymatic digestion with KpnI-HF (New England Biolabs, Ipswich, MA, USA).

### 2.8. Analysis of CRISPR/Cas9 Off-Targets

The bioinformatics-based tool COSMID (CRISPR Off-target Sites with Mismatches, Insertions, and Deletions, https://crispr.bme.gatech.edu/, accessed on 14 September 2022) [53] was used to generate a list of predicted off-target sites. Genomic DNA from modified cells was extracted and used as the template for PCR with primers flanking the predicted off-target sites. The amplicons were sequenced, and TIDE was used to analyze the double-strand break.

## 3. Results

### 3.1. Guide RNA Design, Cloning, and Selection

**Figure 1:**
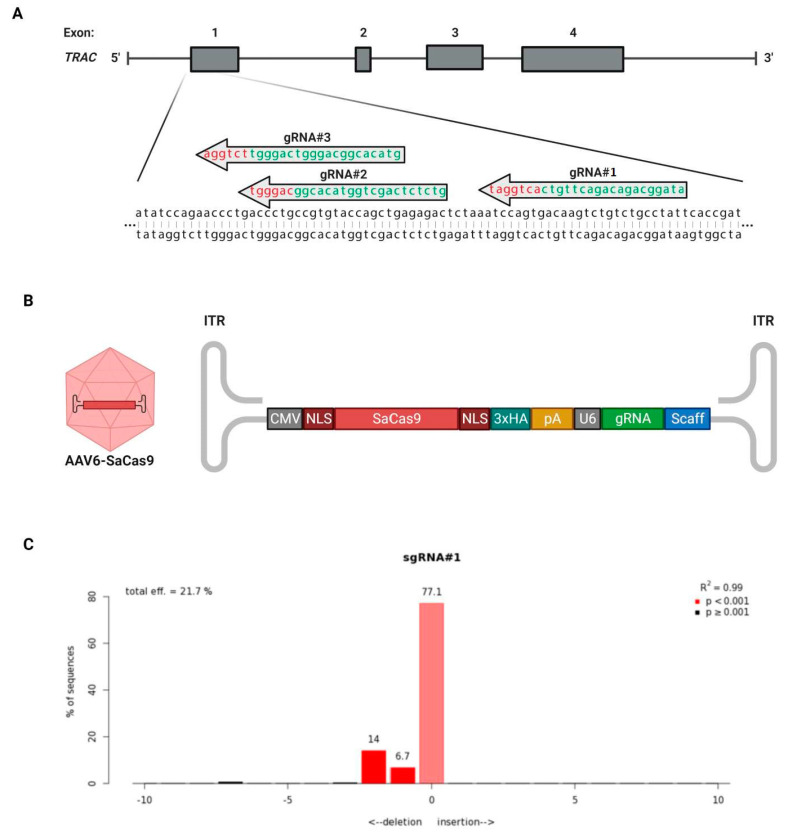
Targeting of *TRAC* locus via AAV6-delivered SaCas9. (**A**) Location of the three designed guide RNAs (gRNAs, in green) and their respective PAM sequences (in red) for targeting of the first exon of the T cell receptor alpha constant (*TRAC*) locus. The four exons of the *TRAC* locus are identified by the numbers 1 to 4. (**B**) AAV-packaged genome encoding for the SaCas9 and one gRNA against the *TRAC* locus. CMV, cytomegalovirus promoter and enhancer; NLS, nuclear localization signal (5′: from SV40 (simian virus 40) large T antigen; 3′: from nucleoplasmin); SaCas9, Cas9 endonuclease from the *Staphylococcus aureus* type II CRISPR/Cas system; 3xHA, three tandem HA epitope tags; pA, bovine growth hormone polyadenylation signal; U6, U6 promoter; gRNA, guide RNA targeting the *TRAC* locus; Scaff, guide RNA scaffold for the *S. aureus* CRISPR/Cas9 system; ITR, inverted terminal repeat. (**C**) Assessment of genome editing by sequence trace decomposition (TIDE) of gRNA #1. Different bar colours indicate different *p*-values: bright red, *p* < 0.001; black, *p* ≥ 0.001.

Three gRNAs were designed to target the first exon of the *TRAC* locus (Figure 1A). Single-strand DNA oligos with complementing sequences corresponding to these gRNAs and a BsaI overhang were synthesized. The complementing strands were annealed and used as templates for cloning into the BsaI site of a plasmid encoding the *Staphylococcus aureus* Cas9 (SaCas9) and the gRNA scaffold flanked by AAV inverted terminal repeats (ITRs) for packaging into AAV vectors (Figure 1B).

The first step was identifying the most efficient gRNA for the targeting and performance of the double-strand break of the *TRAC* locus. For this, the highly permissible cell line HEK293SF was transfected with the plasmids encoding the SaCas9 and one of the gRNAs. Genomic DNA was extracted from the cells 96 h post-transfection, and the CRISPR/Cas9 target site was amplified via PCR. The material was sequenced, and the efficiency of the double-strand break was analyzed by TIDE (Table 1 and Figure 1C). The plasmid containing gRNA #1 (pX601-AAV-SaCas9-TRAC#1) resulted in the highest efficiency (21.7%) and was selected to be used in the following experiments.

### 3.2. Evaluation of Viral Delivery in HEK293SF Cells

**Figure 2:**
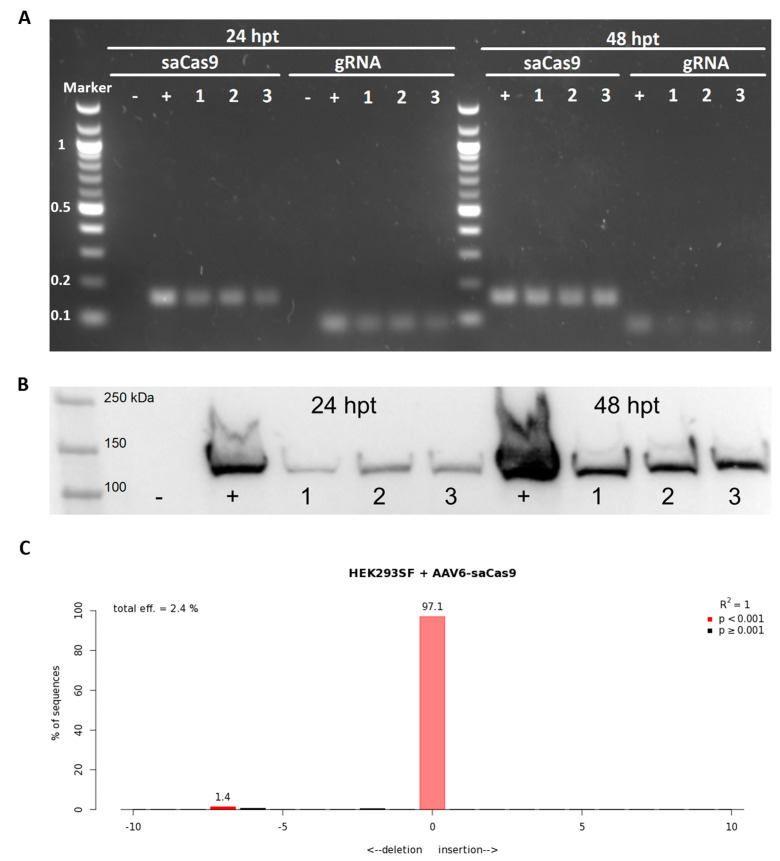
Evaluation of AAV6 delivery of CRISPR/Cas9 system in HEK293SF cells. Samples were collected 24 or 48 h post-transduction (hpt). Negative (−) control is a sample from non-transduced cells; positive (+) control is a sample from cells transfected with a plasmid encoding for SaCas9 and gRNA (pX601-AAV-SaCas9-TRAC#1); 1–3 indicate the replicates. (**A**) Expression of SaCas9 messenger RNA and gRNA detected via agarose gel electrophoresis after RT-PCR. Marker in kb. (**B**) Detection of SaCas9 protein by Western blot. (**C**) Assessment of genome editing by sequence trace decomposition. Different bar colours indicate different *p*-values: bright red, *p* < 0.001; black, *p* ≥ 0.001.

The adeno-associated viral vector packaging the SaCas9 and gRNA #1 (subsequently referred to as AAV6-SaCas9) was produced via the triple transient transfection of HEK293SF cells, purified via affinity chromatography, and concentrated using 100-kDa centrifugal filter units. We first verified the ability of the AAV6-SaCas9 to deliver the CRISPR/Cas9 system and successfully cut the genome at the target site. HEK293SF cells were transduced with AAV6-SaCas9 (MOI ~ 10^3^) in the presence of a human adenovirus serotype 5 (Ad5) at MOI 5. The Ad5 was added to the transduction assay to accelerate and enhance transgene expression [54,55,56]. At 24 and 48 h post-transduction, it was possible to detect the expression of the SaCas9 mRNA and gRNA via agarose gel electrophoresis, with band sizes corresponding to the expected PCR amplicon, respectively 166 and 90 bp (Figure 2A). The Western blot from cell lysate samples showed that the SaCas9 protein was successfully expressed in the cells transduced with AAV6-SaCas9 (Figure 2B). The next step evaluated the DSB efficiency in genomic DNA extracted from the cells 48 h post-transduction. The target site at the *TRAC* locus was amplified via PCR, and sequencing data were analyzed via TIDE (Figure 2C). Differently from the transfection of HEK293SF cells with pX601-AAV-SaCas9-TRAC#1, the transduction with AAV6-SaCas9 resulted in 2.4% indel efficiency, which could be explained by the MOI of AAV6 used.

### 3.3. Design of the DNA Template for Homology-Directed Repair

**Figure 3:**
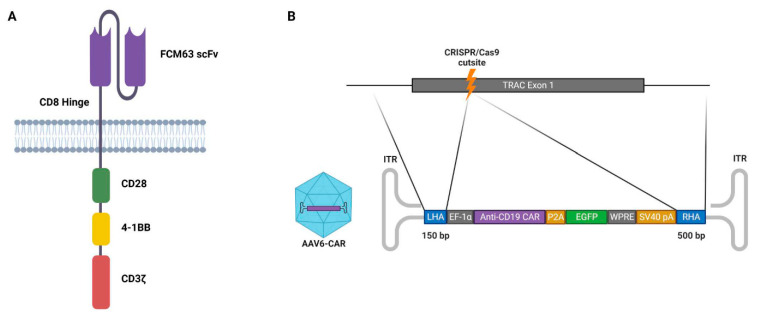
Anti-CD19 chimeric antigen receptor. (**A**) Third-generation chimeric antigen receptor comprising FCM63 single-chain variable fragment (scFv) specific for CD19, CD8 hinge, co-stimulatory domains CD28 and 4-1BB, and signalling domain CD3ζ. (**B**) Top, *TRAC* locus exon 1; bottom, AAV6 containing the CAR cassette flanked by homology arms. LHA, 150-bp left homology arm; EF-1α, core promoter for human elongation factor EF-1α; anti-CD19 CAR, third-generation anti-CD19 chimeric antigen receptor; P2A, 2A self-cleaving peptide; EGFP, enhanced green fluorescent protein; WPRE, woodchuck hepatitis virus posttranscriptional regulatory element; SV40_PA, SV40 (simian virus 40) polyadenylation signal; RHA, 500-bp right homology arm; ITR, inverted terminal repeat.

The third-generation anti-CD19 chimeric antigen was chosen to be incorporated into the DNA template for homology-directed repair. This CAR contains the variable region sequences of the FMC63 monoclonal antibody, which recognizes human CD19 and contains both the CD28 and 4-1BB co-stimulatory signalling domains (Figure 3A). The DNA template also incorporates an EGFP marker and homology arms to the *TRAC* locus (Figure 3B). The total length of the template DNA was 4.0 kb, which allowed sufficient space for the addition of the AAV ITRs. The whole construct was synthesized and cloned into the pUC57 plasmid (pUC57_AAV_anti-CD19_CAR_HDR). This plasmid was used for packaging the DNA template into the AAV6 vectors (subsequently referred to as AAV6-CAR).

### 3.4. Production of Adeno-Associated Viral Vectors and Site-Specific Modification of Jurkat Cells via Dual-AAV Transduction

In order to obtain a sufficient amount of viral vectors for the dual-AAV transduction of the Jurkat cells, a 2-L production was carried out for each vector (AAV6-SaCas9 and AAV6-CAR). The cells, which contained the viral vectors, were pelleted and concentrated by a factor of 4 before viral harvest. The viral titers obtained, expressed as genome-containing viral particles, were 7.7 × 10^9^ and 1.5 × 10^10^ VG/mL for AAV6-SaCas9 and AAV6-CAR, respectively. These viral titers were incompatible with the high-MOI transduction of the Jurkat cells; thus, the viral vector preparations underwent purification via affinity capture chromatography and concentration through a 100-KDa cut-off centrifugal filter unit. As shown in Table 2, the overall vector recovery was around 40–50%. Post-concentration, the viral titer was 1.7 × 10^12^ and 3.9 × 10^12^ VG/mL for AAV6-SaCas9 and AAV6-CAR, respectively. These post-concentration titers were compatible with high-MOI transduction, and the viral vectors were used in the subsequent experiments.

**Figure 4:**
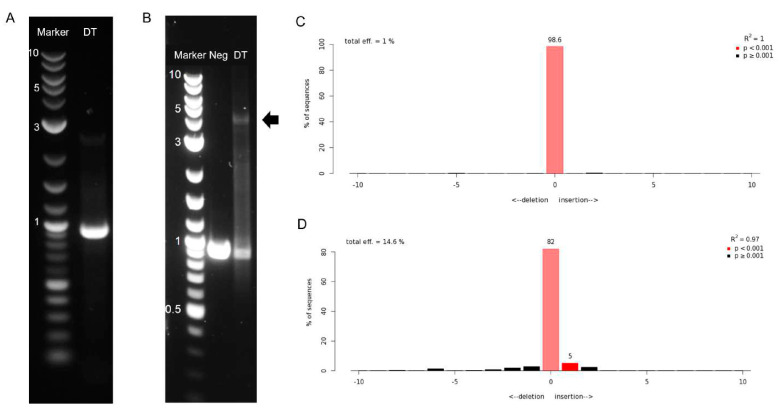
Insertion of anti-CD19 CAR into the *TRAC* locus via dual AAV6 transduction. (**A**) Detection of transgene insertion is not detected in medium-MOI transduction 48 h post-transduction. (**B**) Detection of transgene insertion via PCR amplification of the *TRAC* locus 96 h post-high-MOI transduction. DNA marker in kilobases (kb); Neg, non-transduced Jurkat cells; DT, dual transduced Jurkat cells. Black arrow indicates amplicon with the expected transgene size (~4.3 kb). (**C**) Assessment of genome editing by sequence trace decomposition of medium-MOI transduction. (**D**) Assessment of genome editing by sequence trace decomposition of high-MOI transduction. Different bar colours indicate different *p*-values: bright red, *p* < 0.001; black, *p* ≥ 0.001.

The dual-transduction of Jurkat cells (Figure 4) was first conducted at a medium MOI, where the cells were transduced with both AAV6 vectors at an MOI of 10^5^ VG/cells. The transgene insertion was evaluated via analysis of the gDNA 48 h post-transduction; however, it was not possible to detect the integration in the target locus (Figure 4A), and a TIDE analysis of the sequencing traces resulted in a total DSB efficiency of 1% (Figure 4C). Molecular analysis of the target locus did not result in the detection of transgene integration either. The low efficiency in the DSB and failure to detect transgene integration could have resulted from inefficient transduction due to a still not appropriate MOI or a premature analysis of the genomic modification. The incubation time of only 48 h may have been insufficient for the formation of the complementary second strand of the single-stranded AAV genome, which is needed for the expression of the transgene. It is plausible that this incubation was too brief to allow for the expression of the nuclease, which is essential for genome cleavage. Therefore, a longer incubation period may be necessary.

A second transduction was conducted at a high MOI to account for the previously observed problems. AAV6-SaCas9 and AAV6-CAR were added to the cells at MOI 2 × 10^6^ and 7 × 10^6^ VG/cells, respectively. Furthermore, the genomic analysis was done 96 h post-transduction, allowing more time for the expression and function of the nuclease, increasing the possibility of detecting both genomic cleavage and homology-directed repair. Following a PCR using primers flanking the target region, a band corresponding to the expected size of the inserted transgene (4.3 kb) was detected via agarose gel electrophoresis (Figure 4B). This band was calculated to have a relative abundance of 10.9% via densitometric analysis. Moreover, the absolute quantitation of the bands resulted in an abundance of 9.1% for the 4.3-kb band. The TIDE analysis of this high-MOI dual transduction resulted in 14.6% of DSB efficiency (Figure 4D). It is important to note that TIDE can only detect small indels of up to 50 base pairs. Thus, this result does not consider transgene insertion via homology-directed repair. These results suggest that approximately 10% of the genomic material analyzed contained the insertion.

**Figure 5:**
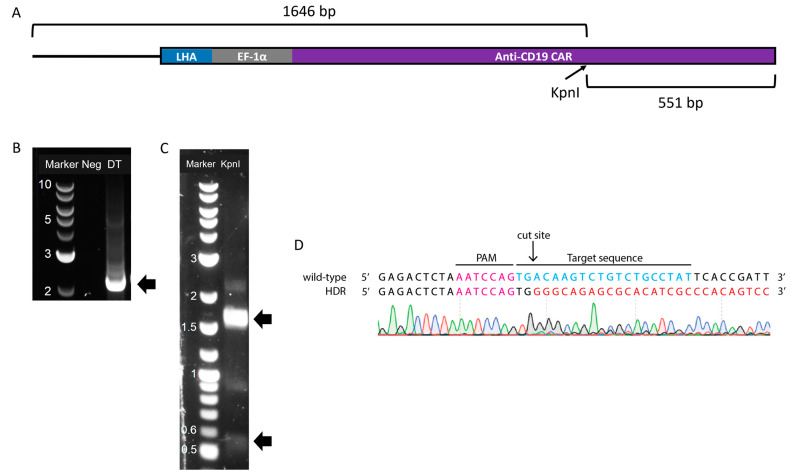
Molecular analysis of site-specific homology-mediated knock-in of anti-CD19 CAR gene. (**A**) Schematic representation of the PCR product containing the inserted DNA template with a KpnI site located within the CAR gene. (**B**) Agarose gel electrophoresis of the amplicons from non-transduced cells (Neg) and dual-transduced (DT) cells, resulting in a DNA band of 2.2 kb (black arrow). (**C**) Agarose gel electrophoresis of the 2.2-kb fragment digested with KpnI. Black arrows indicate the expected fragments with sizes 1.6 and 0.5 kb. The 2.2-kb band from the undigested material is also visible. DNA marker in kilobases (kb). (**D**) Sequence alignment of the 5′-end of genome-edited cells. Magenta, PAM site; blue, gRNA binding site; and red, integrated donor fragment.

To further confirm the insertion of the transgene into the specific CRISPR/Cas9 cut site, the amplified product from the TRAC-flanking PCR was purified and used as the template for a second PCR (“nested”). A nested PCR was done using the same forward primer flanking the *TRAC* locus and a reverse primer that annealed inside the transgene, more specifically in the CAR region, resulting in the product shown in Figure 5A. As expected, a band of 2.2 kb was obtained (Figure 5B). This band was then extracted and digested with KpnI, generating approximately 550- and 1650-bp (Figure 5C) fragments corresponding to the expected digested DNA. The 2.2-kb band from the undigested fragment was also visible, as well as a 0.9-kb band, the same size as the unmodified *TRAC* locus also visible in Figure 4B. The material from the 2.2-kb band was also sequenced, confirming the insertion of the transgene template at the *TRAC* locus (Figure 5D).

### 3.5. Off-Target Evaluation

Finally, to evaluate the specificity of the chosen gRNA, COSMID detected 22 sites in the genome that diverged from the expected target site by less than four base pairs. The predicted sites were ranked based on similarity to the target sequence. Table 3 shows six of the top predicted sites evaluated for the occurrence of double-strand breaks in transfected HEK293SF cells and the dual-transduced Jurkat cells. Indel frequencies were low for all the potential off-target sites. In the HEK293SF cells, the off-target sites where activity was the highest (OT1 and OT6) were mutated in 1.5% of the cells; however, they were not located in known gene coding regions. The second-highest indel frequency (OT4, 1.3%) was detected in the gene *CACBN2*. The third-highest indel frequency was detected in a pseudogene (*LPAL2*). Three off-target sites failed to be sequenced for the indel analysis in Jurkat cells (OT1, OT4, and OT10), due to inadequate template quality/concentration or due to the presence of double sequences. No indel was identified for the remaining analyzed sites, corresponding to OT3, OT6, and OT8. These results show that the SaCas9 coupled with the chosen gRNA induces DNA breaks at the TRAC locus with a high specificity profile and without significant off-target effects.

## 4. Discussion

To avoid the use of the Cas9 from *Streptococcus pyogenes* (SpCas9) and, consequently, having to split the CRISPR/Cas9 machinery into two AAV vectors [57,58], we decided to utilize the smaller Cas9 from *Staphylococcus aureus* (SaCas9) in order to package both nuclease and gRNA into a single AAV vector [49]. The SaCas9 efficiency is comparable to that of SpCas9, and it is able to mediate in vivo genome editing [59,60,61]. Moreover, depending on the PAM sequence, SaCas9 presents higher cleavage activity than SpCas9 and FnCpf1 [62]. From the three gRNAs targeting the first exon of the *TRAC* locus tested in this study, we achieved gene editing efficiency similar to results previously reported for other targets and cell types [61].

The AAV serotype 6 was chosen due to its high efficiency in transducing T cells compared to other serotypes [44]. However, the MOI required to effectively transduce cells is considered high, reaching values around 10^6^ VG/cell [42,44]. This could explain the low indel efficiencies obtained when the CRISPR/Cas9 machinery was delivered via AAV6 at a low MOI to HEK293SF and medium MOI to Jurkat cells. The genome modification efficiency increased significantly when Jurkat cells were transduced at a high MOI, as reported by other researchers [63,64].

The DNA template design had to consider the ~4.7-kb packaging limit of the AAV vector. The transgene containing the anti-CD19 CAR and EGFP marker spanned over 3.4 kb, with the ITRs accounting for an additional 400 bp. This implies around 900 bp in designing the homology arms. We then decided to evaluate the shortest homology arms necessary for a successful HDR, as determined by Hirata and Russell [65]. Thus, the homology arm upstream of the transgene (left homology arm, LHA) contained a 150-bp homologous to nucleotide sequence upstream of the cut site in the *TRAC* locus. Downstream of the transgene, a 500-bp homology arm was included. Both the MOI and the length of the homology arms are essential factors for the effectiveness of gene targeting by AAV vectors [16,65]. Homology arms ranging from 300 to 985 bp have been described for the AAV6-mediated targeting of CAR into the *TRAC* locus [46,47] and around 600 bp for other genomic locations [45,48]. The HDR efficiency obtained in our study (~10%) is similar to the one reported by Sather et al. [45] while using megaTAL, but slightly inferior (2–5 times lower) to those achieved with homing nucleases or CRISPR [20,36,46]. Future studies should optimize the MOI and the length and position of the homology arms in order to improve the HDR efficiency obtained in this study and to allow the future use of these vectors in a therapeutic setting [44]. Increasing the length of the homology arms would be possible while still within the AAV packaging limit by removing the fluorescent marker, EGFP, or the exogenous promoter, EF1-α, from the transgene cassette. Fluorescent markers are helpful during pre-clinical research but are not desired for clinical applications. By removing the exogenous promoter and controlling the expression of the CAR transgene by the endogenous *TRAC* promoter, stable and robust expression can be achieved, similar to the physiological TCR [34]. 

In this work, we report the effective combination of CRISPR and AAV6 delivery of a DNA template for the targeted insertion of CAR into the *TRAC* locus. Similar methods have been published previously for the site-specific integration of CAR [20,36,48]. In these reports, however, the CRISPR machinery is delivered as ribonucleoproteins (RNPs) via electroporation of the cells, which limits their approaches to in vitro or ex vivo genome modification. Because our method exploits an AAV6 vector to deliver the CRISPR/Cas9 machinery, it opens up the possibility for the in vivo generation of CAR-T cells. In vivo delivery of RNPs can be achieved through the use of lipid-based nanocarriers [66], polymer-based nanoparticles [67,68], peptide nanoparticles [69,70], or inorganic nanoparticles [71]. These methods, however, have limited efficiency compared to AAV vectors [72]. In vivo delivery of CRISPR-based therapies via AAV vectors has been reported in several research types with wildtype and disease-model animals [73]. Nawaz et al. [74] recently reported the AAV-mediated in vivo generation of CAR-T cells. In addition, in mice, dual-AAV delivery has been successfully reported for the in vivo HDR-mediated correction of metabolic liver disease and modification of mitotic and postmitotic neurons [64,75].

Low cell recovery was observed after 48 h from the transduction, which prevented us from confirming the expression of the CAR transgene inserted into the TRAC locus. This could have resulted from genotoxicity due to the high MOI needed for efficient transduction [76]. Further investigation is necessary to increase the recovery of the modified cells. Other studies have employed a similarly high MOI without reporting toxicities; however, different transduction regimens were used [20,44,45]. For instance, Wang et al. [44] replaced the culture medium 16 h after adding the viral vectors to halt the transduction. On the other hand, we decided to maintain the viral vector in the culture medium in an attempt to enhance transduction and further improve the chances for genome modification. Other approaches to improve cell recovery include the optimization of the seeding density, medium supplementation with higher amounts of fetal bovine serum, supplementation with IL-2, and/or cell activation via CD3 and CD28 receptors [77]. Another possible cause of the observed toxicity is off-target genome modification by the CRISPR/Cas9 system. However, only low percentages of off-target effects were identified. Most occurred in low-risk locations, such as non-coding sequences or pseudogenes. The only off-target identified in a known gene occurred in *CACBN2*, which is dominantly expressed in the retina (https://www.proteinatlas.org/ENSG00000165995-CACNB2, acceded on 17 February 2023) and should have a limited impact in T cells. The SaCas9 has been characterized as inducing very low percentages of off-target indels [49]. However, it is not possible to disregard that a non-identified off-target resulted in decreased cell viability over time. Hence, a deeper screening of off-target activity is essential in improving the safety and efficacy of the method here presented [73].

To ensure a thorough understanding of the effectiveness of the dual-AAV6 transduction method, it is necessary to conduct further analyses to evaluate and characterize the modified cells. This includes examining the expression of the CAR protein and the endogenous TCR on the surfaces of the transduced cells through flow cytometric analysis. Successful CAR integration into the *TRAC* locus should replace the expression of the endogenous TCR with the anti-CD19 CAR. These two proteins can also be used for cell sorting and enrichment of the culture for TCR^−^CAR^+^ cells. Evaluating the functionality of anti-CD19 CAR-T cells can be achieved by co-cultivating them with CD19-expressing target cells. Although Jurkat cells cannot induce the death of target cells, they are activated by the target antigen, which can be detected by the expression of CD69 on the Jurkat cell surface [50]. Furthermore, conducting a complete immunophenotypic analysis of the CAR-T cells can provide a better understanding of the cells’ phenotype, activation, and functional profile [78].

## 5. Conclusions

These results serve as a proof of concept that dual-AAV6 transduction can be employed for the site-specific integration of anti-CD19 CAR into T cells, with no significant off-target effects. Potential applications of the method reported here include the generation of ex vivo “off-the-shelf” universal immunotherapy. Further research is needed to improve the HDR efficiency, assess CAR protein expression and TCR knockout, and evaluate CAR functionality against target cells. This dual-AAV transduction process broadens the possibility for the in vivo generation of CAR-T cells but is not limited to this transgene and could be exploited for other gene therapy applications.

## Figures and Tables

**Table 1:** Double-strand break efficiency of tested guide RNAs.

gRNA Sequence	Efficiency (%)
#1 ataggcagacagacttgtca	21.7
#2 gtctctcagctggtacacgg	12.1
#3 tacacggcagggtcagggtt	2

**Table 2:** Production of AAV6 vectors.

Vector	Step	Total VG	% Step Recovery	% Overall Recovery
AAV6-SaCas9	Harvest	4.34 × 10^12^	100	100
	Purification	1.91 × 10^12^	44.08	-
	Concentration	1.76 × 10^12^	91.75	40.47
AAV6-CAR	Harvest	8.65 × 10^12^	100	100
	Purification	8.17 × 10^12^	94.40	-
	Concentration	3.99 × 10^12^	48.89	46.15

**Table 3:** Analyzed off-target sites.

Name	Target Sequence	Gene	Product	% Indel	
				HEK293SF	Jurkat
***gRNA #1***	*ATAGGCAGACAGACTTGTCACTGGAT*	*TRAC*	*T-cell receptor*	*21.7*	*14.6*
**OT1**	AAGTAAGACAGACTTGTCAGTGGGT	LOC112267962	Long non-coding RNA	1.5	-
**OT3**	ATAGTCACACAGACTGTCACTGAAT	LINC01035	Long non-coding RNA	0.1	0
**OT4**	ATAGGAGACAGGATTGTCAAGGGAT	CACNB2	Voltage-gated calcium channel	1.3	-
**OT6**	ATAGAGCAGACAGACAGGTCAAAGAAT	-	-	1.5	0
**OT8**	ATGGCAGAGAGACTTGCCATTGGAT	LPAL2	Lipoprotein(a) 2 pseudogene	1.1	0
**OT10**	GTAGTCAGACAGACTTGTATTGGAT	GTDC1	Glycosyltransferase-like	0.2	-

Analysis of desired target site is italicized.

## Data Availability

The original contributions presented in the study are included in the article; further inquiries can be directed to the corresponding author.
